# Supplementary material for: Phase-stabilised self-injection-locked microcomb
Source: Nat Commun. 2024 Aug 15;15:7030. doi: 10.1038/s41467-024-50842-8 (PMC11327250; doi:10.1038/s41467-024-50842-8)
Supplement: Supplementary file 1 — Supplementary Information [file 41467_2024_50842_MOESM1_ESM.pdf]

Supplementary Information - Phase-stabilised self-injection-locked microcomb

Thibault Wildi[1,†], Alexander Ulanov[1,†], Thibault Voumard[1], Bastian Ruhnke[1], Tobias Herr[1,2,*]

[1]Deutsches Elektronen-Synchrotron DESY, Notkestr. 85, 22607 Hamburg, Germany
[2]Physics Department, Universität Hamburg UHH, Luruper Chaussee 149, 22761 Hamburg, Germany
[†]These authors contributed equally
[*]tobias.herr@desy.de

1 Tuning of the offset frequency f_{off}

1.1 Diode laser current

The tuning rate of the diode laser frequency used in our work is approximately $1\,\mathrm{GHz\,mA^{-1}}$. As illustrated in Figure S1, self-injection locking (SIL) reduces this tuning rate by a factor $\sigma = \partial\xi/\partial\zeta$ due to the feedback from the microresonator. In our case, we estimate $\sigma \approx 40$ when operating in the SIL dissipative Kerr soliton (DKS) regime [1] (the exact value of σ depends on the detuning between diode laser and microresonator), and therefore

$$\frac{\partial f_{\mathrm{off}}}{\partial I_{\mathrm{p}}} \approx 25\,\mathrm{MHz\,mA^{-1}}\,. \tag{1}$$

This estimate is in excellent agreement with the measured value of $27\,\mathrm{MHz\,mA^{-1}}$ (c.f. Table 1 in manuscript). Furthermore, we note that SIL reduces the diode laser's linewidth by a factor σ^2, often known as the SIL stabilisation coefficient. This more than 1000-fold reduction in linewidth significantly reduced the bandwidth requirement for the actuators and is critical in enabling phase-locking via heater control.

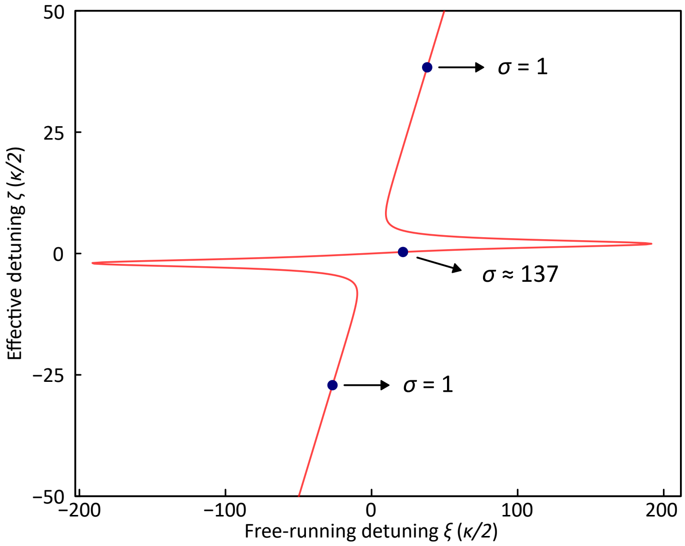

Figure S1 | **Conceptual illustration of a SIL tuning curve**. The effective detuning $\zeta = 2(\omega_0 - \omega_e)/\kappa$ is shown as a function of the laser-microresonator detuning $\xi = 2(\omega_0 - \omega_{LC})/\kappa$ (ω_0 is the resonance frequency of the microresonator, ω_e the laser emission frequency and ω_{LC} the free-running laser frequency, i.e. the laser's emission frequency in the absence of SIL). Outside of the SIL range, there is a one-to-one dependence between ζ and ξ, whereas, in SIL operation, the laser tuning speed $\partial\zeta/\partial\xi$ is reduced by a factor σ.

1.2 Heater current

Similarly, varying the microheater current modifies the temperature of the microresonator and hence affects the diode laser's emission frequency through the SIL mechanism. In our system, we measure the heater tuning coefficient to be approximately $1\,\mathrm{GHz\,mW^{-1}}$ in our chips (thermally-induced frequency shift per unit of dissipated electrical power), which corresponds to

$$\frac{\partial f_{\mathrm{off}}}{\partial I_{\mathrm{h}}} \approx 210\,\mathrm{MHz\,mA^{-1}} \tag{2}$$

at our operation point (current bias of $3\,\mathrm{mA}$). This is in good agreement with the measured value of $160\,\mathrm{MHz\,mA^{-1}}$ (c.f. Table 1 in manuscript).

2 Tuning of the repetition rate f_{rep}

To quantify the repetition rate tuning, we need to include the Raman-induced soliton self-frequency shift (SFS) in our considerations. The SFS is given by [2, 3]:

$$\Omega \approx -\frac{64\pi^2}{15}\frac{D_1^2}{2\pi D_2}\delta f_{\mathrm{R}}\tau_{\mathrm{R}}\,, \tag{3}$$

where D_1 and D_2, describe the microresonator's mode frequencies $\omega_\mu = \omega_0 + D_1\mu + D_2/2\mu^2$ (μ is the relative mode index), $\delta = (\omega_0 - \omega_{\mathrm{p}})/(2\pi)$ the pump to resonance detuning, and f_{R} and τ_{R} the Raman fraction and shock terms respectively, taken to be 20 % and 20 fs in silicon nitride [2]. The SRS translates directly to a change in the repetition rate via the cavity dispersion:

$$\Delta f_{\mathrm{rep}} = \frac{\Omega}{2\pi}\frac{D_2}{D_1} \approx D_1\delta f_{\mathrm{R}}\tau_{\mathrm{R}}\,. \tag{4}$$

Hence, the repetition rate is sensitive to the detuning δ via the Raman-induced SFS.

2.1 Diode laser current

As discussed in Section 1.1, the detuning δ can be tuned through the diode laser current at a rate of $25\,\mathrm{MHz\,mA^{-1}}$. Using eq. 4, we find

$$\frac{\partial f_{\mathrm{rep}}}{\partial I_{\mathrm{p}}} \approx 190\,\mathrm{kHz\,mA^{-1}}, \tag{5}$$

which is in good agreement with the measured value of $160\,\mathrm{kHz\,mA^{-1}}$ (c.f. Table 1 in manuscript).

2.2 Heater current

The detuning δ can also be adjusted via the heaters, although the tuning rate is reduced through the SIL mechanism by σ to approximately $5\,\mathrm{MHz\,mA^{-1}}$. Again using eq. 4, we find

$$\frac{\partial f_{\mathrm{rep}}}{\partial I_{\mathrm{h}}} \approx 38\,\mathrm{kHz\,mA^{-1}}. \tag{6}$$

On the other hand, thermal actuation directly affects the ring's FSR (via the thermorefractive effect and to a lesser extent, through thermal expansion) at a rate

$$\frac{1}{2\pi}\frac{\partial D_1}{\partial I_{\mathrm{h}}} \approx 325\,\mathrm{kHz\,mA^{-1}}. \tag{7}$$

These two different contributions add up to about $\partial f_{\mathrm{rep}}/\partial I_{\mathrm{h}} = 360\,\mathrm{kHz\,mA^{-1}}$, which is again in good agreement with the measured value of $400\,\mathrm{kHz\,mA^{-1}}$ (c.f. Table 1 in the main manuscript).

3 Actuator linearity

The dependence of the microcomb repetition rate f_{rep} and offset frequency f_{off} where recorded as a function of the diode laser current I_{p} and heater current I_{h} (see Figure S2). All tuning curves are monotonic and, to good approximation, linear, which ensures stable locking conditions.

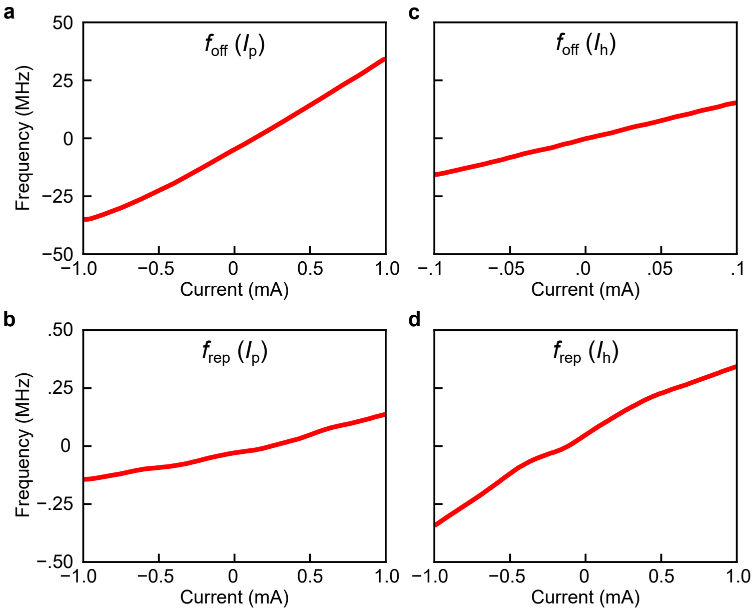

Figure S2 | **Actuator tuning curves**. Values are shown around the system's operating point. **a**, Offset frequency f_{off} as a function of the diode laser current I_{p}. **b**, Repetition rate frequency f_{rep} as a function of the diode laser current I_{p}. **c**, Offset frequency f_{off} as a function of the microheater current I_{h}. **d**, Repetition rate frequency f_{rep} as a function of the microheater current I_{h}.

4 Microheater frequency response

We measure the open-loop microheater frequency response by using a slow side-of-fringe lock to stabilise a continuous wave laser on a resonance of the microresonator. A sinusoidal tone is then applied to the microheater, which modulates the resonance frequency and, thereby, the transmission of the continuous wave laser. We can extract the microheater's frequency response by recording the amplitude and phase relation between the input and output signal (see Figure S3).

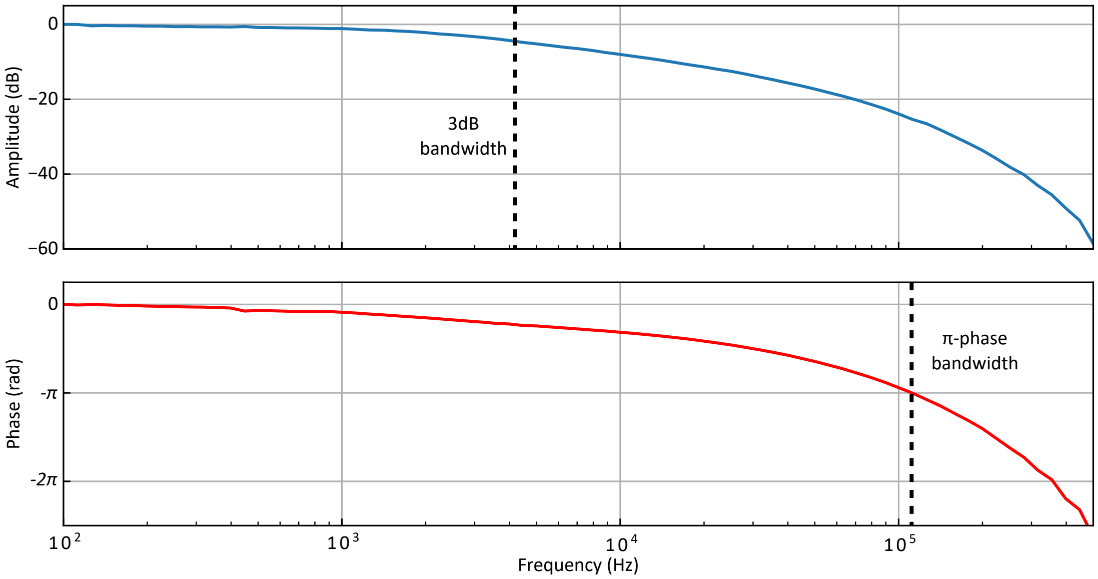

Figure S3 | **Open-loop microheater frequency response.** Amplitude (top) and phase (bottom) frequency response of the micro-heater. While the 3 dB bandwidth is approximately 5 kHz a phase delay of π is reached at $\sim$100 kHz.

As can be seen, the 3 dB of the microheater is approximately 5 kHz. Despite this, a phase delay of π is reached at $\sim$100 kHz. This allows us, in a closed-loop configuration, to effectively extend the heaters's bandwidth to more than 100 kHz. As the heater bandwidth dictates the overall system bandwidth, this step is critical in achieving both offset and repetition rate locks.

5 Comparison of fully phase-locked microcombs

A comparison of fully phase-locked microcombs to date (i.e. ν_p and f_rep are both phase-coherently stabilised to an external frequency reference) is provided in Table 1, where we list resonator platform, form factor, the employed actuators as well as important characteristics such as pump laser power, electrical power consumption, volume (excluding driving electronics) and approximate unit cost.

**Ref.**	**Platform**	**Form factor w/ actuators**	**1st actuator**	**2nd actuator**	**Pump [a] (mW)**	**Elec.[b] (W)**	**Volume[c] (cm^3)**	**Cost[d] (USD)**
[4]	SiO_2 WGM	Table-top	ECDL detuning	EDFA power	200	>10	>1000	>10000
[5]	MgF_2 WGM	Table-top	FL detuning	Aux. FL detuning	240	>10	>1000	>10000
[6]	SiO_2 WGM	Table-top	ECDL detuning	EDFA power	100	>10	>1000	>10000
[7]	Si_3N_4 PIC	Table-top	ECDL detuning	AOM power	2000	>10	>1000	>10000
[8]	Si_3N_4 PIC	Table-top	SSB modulator	ECDL/EDFA power	200	>10	>1000	>10000
This work	Si_3N_4 PIC	Chip-scale	SIL DFB current	Microheater	25	<0.5	<1	$\lesssim 100$

WGM, whispering-gallery-mode resonator; PIC, photonic integrated circuit microring resonator; ECDL, external cavity diode laser; EDFA, erbium-doped fibre amplifier; FL, fibre laser; AOM, acousto-optic modulator; SSB single sideband; SIL, self-injection locking; DFB, distributed feedback laser diode. [a]Coupled optical pump power. [b]Estimated electrical power consumption of the optical frequency comb source. [c] Estimated volume excluding driving electronics. [d]Estimated unit cost, when fabricated at scale.

Table 1 | Comparison of fully phase-locked microresonator frequency combs.

References

[1] Voloshin, A. S. *et al.* Dynamics of Soliton Self-Injection Locking in Optical Microresonators. *Nature Communications* **12**, 235 (2021).

[2] Karpov, M. *et al.* Raman Self-Frequency Shift of Dissipative Kerr Solitons in an Optical Microresonator. *Physical Review Letters* **116**, 103902 (2016).

[3] Yi, X., Yang, Q.-F., Yang, K. Y., and Vahala, K. Theory and Measurement of the Soliton Self-Frequency Shift and Efficiency in Optical Microcavities. *Optics Letters* **41**, 3419 (2016).

[4] Del'Haye, P., Arcizet, O., Schliesser, A., Holzwarth, R., and Kippenberg, T. J. Full Stabilization of a Microresonator-Based Optical Frequency Comb. *Physical Review Letters* **101**, 053903 (2008).

[5] Jost, J. D. *et al.* All-Optical Stabilization of a Soliton Frequency Comb in a Crystalline Microresonator. *Optics Letters* **40**, 4723–4726 (2015).

[6] Del'Haye, P. *et al.* Phase-Coherent Microwave-to-Optical Link with a Self-Referenced Microcomb. *Nature Photonics* **10**, 516–520 (2016).

[7] Brasch, V. *et al.* Photonic Chip–Based Optical Frequency Comb Using Soliton Cherenkov Radiation. *Science* **351**, 357–360 (2016).

[8] Briles, T. C. *et al.* Interlocking Kerr-microresonator Frequency Combs for Microwave to Optical Synthesis. *Optics Letters* **43**, 2933 (2018).
